# Physiological Changes and Transposition of Insertion Sequences in the *dps*-Double-Knockout Mutant of *Deinococcus geothermalis*

**DOI:** 10.3390/ijms27031238

**Published:** 2026-01-26

**Authors:** Yujin Park, Hyun Hee Lee, Eunjung Shin, Soyoung Jeong, Sung-Jae Lee

**Affiliations:** 1 Department of Biology, College of Sciences, Kyung Hee University, Seoul 02447, Republic of Korea; sune1112@khu.ac.kr (Y.P.); heemoon45@naver.com (H.H.L.); eunj@khu.ac.kr (E.S.); syjeong1010@gmail.com (S.J.); 2 Korea Institute of Ornithology, Kyung Hee University, Seoul 02447, Republic of Korea

**Keywords:** *Deinococcus geothermalis*, DNA-protecting protein in starved cell (Dps), gamma irradiation, hydrogen peroxide (H_2_O_2_), insertion sequence (IS), oxidative stress, phenotypic selection of non-pigment, transposition

## Abstract

DNA-protecting proteins (Dps) are crucial for safeguarding chromosomal DNA in starved cells during the stationary phase under stressful conditions. In previous research, the two Dps proteins in *Deinococcus geothermalis*, Dgeo_0257 (Dps3) and Dgeo_0281 (Dps1), were found to complement each other in protecting DNA from oxidative damage. This study investigates the physiological changes and transposition of insertion sequences (ISs) in a double-knockout (DK) mutant lacking both *dps* genes. Comparisons between the wild-type and mutant strains revealed significant phenotypic differences in viability under oxidative stress conditions induced by hydrogen peroxide and ferrous ions, particularly during the stationary phase. Notably, oxidative stress triggered the transposition of the IS families IS*701* and IS*5*, with IS*66* being transposed exclusively in the DK mutant into a gene encoding phytoene desaturase. Transcriptomic analysis using RNA-seq revealed substantial fold changes in gene expression across the genome. For example, the *dgeo*_1459–1460 gene cluster, which encodes a DUF421 domain-containing protein and a hypothetical protein, was highly upregulated under both oxidative and non-oxidative conditions. Interestingly, catalase, encoded by a single gene in *D. geothermalis*, was upregulated in the DK mutant during the stationary phase, with expression levels exceeding those observed in the single *dps* gene-deficient mutants. Conversely, a prominent downregulation of the Fur family regulator was detected. These findings highlight the growth phase-dependent physiological adaptation of the *dps*-DK mutant and reveal a novel IS transposition event of the IS*Bst12* group involving the IS*66* family. Therefore, this study provides new observations into the influence of DNA-protective protein deficiency on oxidative stress responses and IS transposition in *D. geothermalis*, as well as the regulatory mechanisms of the catalase induction pathway, raising the need for further investigation into the role of OxyR.

## 1. Introduction

The genus *Deinococcus* is renowned for its exceptional resistance to ionizing radiation (IR), ultraviolet (UV) light, desiccation, and oxidative stress caused by agents such as hydrogen peroxide (H_2_O_2_) and arsenic ions [1,2,3]. This resilience is primarily attributed to highly efficient DNA repair systems and protective mechanisms against environmental stressors [2,4]. Strains of *Deinococcus geothermalis*, a red-pigmented, Gram-positive bacterium with spherical cells (1.5–2 µm in diameter), have been isolated from hot springs in Portugal and Italy, as well as from colored biofilms in paper machines [5,6,7,8].

Among these protective mechanisms, DNA-protecting proteins in starved cells (Dps) are well known to play one of the central roles in managing oxidative stress. Dps, a family of conserved prokaryotic proteins, protect DNA by mitigating the damage caused by hydroxyl radicals generated through the Fenton reaction, which involves H_2_O_2_ and ferrous ions (Fe^2+^) [9,10,11]. They achieve this by (1) sequestering Fe^2+^ ions to prevent hydroxyl radical formation, and (2) directly binding DNA to shield it from oxidative damage [11,12]. In *Escherichia coli*, a model organism, Dps proteins form dodecameric structures capable of storing up to 500 Fe^2+^ ions, thereby maintaining iron homeostasis and preventing damage from reactive oxygen species (ROS). Nevertheless, despite the presence of other established iron-sequestering proteins such as ferritin and bacterioferritin, Dps proteins serve a distinct and indispensable function within *E. coli* [11]. In *E. coli*, Dps proteins function as dominant nucleoid-associated proteins (NAPs) during the stationary phase, surpassing other NAPs such as HU, factor for inversion stimulation (FIS), and histone-like nucleoid-structuring protein (H-NS). This growth phase-dependent role underscores its importance in DNA protection and gene expression during stress and nutrient starvation [13]. A recent study demonstrated that in *E. coli*, IS transposition into the region upstream of the *bgl* operon occurs at a higher rate compared to random mutations, a phenomenon mediated by H-NS [14].

In *Deinococcus*, two Dps proteins, Dps1 and Dps2, have been characterized in *Deinococcus radiodurans* R1. Dps1 protects DNA under oxidative stress via a dodecameric conformation, while the role of Dps2 remains unclear [15,16,17]. In *D. geothermalis*, our previous studies identified two Dps proteins, Dgeo_0281 (Dps1 as a main Dps) and Dgeo_0257 (Dps3 as a putative Dps; Dps2 was omitted in *D. geothermalis* genomes), which exhibit complementary expression patterns across growth phases [18]. Dps1 is upregulated in the early exponential phase, whereas Dps3 predominates during the stationary phase. Notably, the deletion of either gene results in compensatory overexpression of the other [18].

In general, bacterial genomes contain abundant transposable elements, especially various families of insertion sequences (ISs), whose structural diversity has been conceptually and effectively described in terms of size (typically less than 3 Kb), the number of encoded genes, unique sequence elements such as terminal inverted repeats (TIRs), and transpositional mechanisms, based on studies of the mobilome and experimental findings [19,20,21,22,23,24]. IS elements transfer into other loci in genomes through a key enzyme, transposase (Tpase), which is induced by various environmental factors including ultraviolet light, chemicals, gamma irradiation, heat shock, and other oxidative agents, resulting in genomic plasticity primarily through gene disruption and gene activation, with no detectable effect on nearby gene expression [21,22,25,26]. Several IS family members have been characterized in terms of their transpositional mechanisms, which are dependent on physiological and stress response conditions [24,27]. These IS elements have been classified through comparative identification of conserved domains in Tpase, supported by IS analysis platforms, especially ISfinder (https://isfinder.biotoul.fr/, accessed on 5 December 2025) [28].

In previous studies, single *dps*-deficient mutants (Δ*dgeo*_0257 as Δ*dps3* and Δ*dgeo*_0281 as Δ*dps1*) also exhibited IS transposition events [29,30]. For instance, IS*Dge5* and IS*Dge6* were transposed in the Δ*dps3* mutant, while IS*Dge2* was active in both the Δ*dps3* and Δ*dps1* mutants under IR or dielectric barrier discharge (DBD) plasma treatment [31]. IS transposition was confirmed in each single mutant, and building upon previous studies demonstrating the complementary roles of *dps* (genes/proteins), an investigation of IS transposition in the double-knockout (DK) mutant (Δ*dgeo*_0257–0281 as Δ*dps*-DK) is anticipated to provide further information on the conditions influencing IS mobility under oxidative stresses. In this study, we examined the effects of oxidative stress on IS transposition in a Δ*dps*-DK mutant lacking both *dps* genes in *D. geothermalis*. The Δ*dps*-DK mutant showed growth patterns similar to those of the single mutants but exhibited increased sensitivity to H_2_O_2_ in the presence of ferric ions during the stationary phase. IS*Dge4*, a member of the IS*66* family, was found to transpose in this mutant for the first time, along with the IS*701* and IS*5* families. Despite transcriptomic analysis revealing generally subtle changes in gene expression levels, qRT-PCR confirmed the significant regulation of genes associated with oxidative stress responses, including catalase and various transcriptional regulators such as the Fur family, LysR family, and HU, as well as unknown functional gene clusters. Given that insertional events occurring under specific cellular stresses often lead to a phenotype that relieves that stress, our research on the Δ*dps*-DK mutant could also offer clues to explain a related phenomenon. Notably, the pronounced downregulation of the Fur family regulator in the Δ*dps*-DK mutant coincided with significantly increased catalase levels, suggesting that this might be potentially mediated by direct or indirect regulation involving the Fur protein. Thus, this study discusses the potential regulatory networks linking oxidative stress responses to IS transposition in *D. geothermalis*.

## 2. Results

### 2.1. Construction of dps-DK Mutant and Physiological Properties

**Figure 1:**
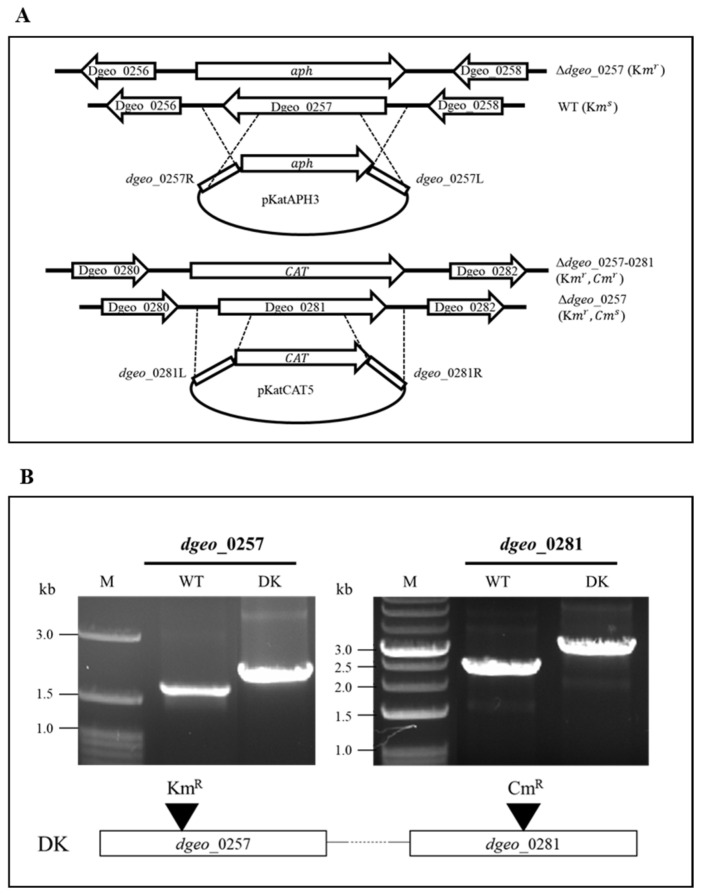
(**A**) Scheme of both *dps* genes as *dps3* and *dps1* double-knockout (DK) mutants using both kanamycin and chloramphenicol antibiotic selection. (**B**) Confirmation of target gene disruption by antibiotic-resistant gene integration through PCR amplification using encompassing primer sets, resulting in enlarged PCR products in Δ*dps*-DK mutant.

To construct the Δ*dps*-DK mutant, a recombinant plasmid containing a chloramphenicol resistance gene flanked by the left and right border regions of *dps1* was transformed into the Δ*dps3* mutant, which has a kanamycin-resistant phenotype. Selection was performed on TGY agar plates containing 3 µg/mL chloramphenicol (Figure 1A). The successful double disruption of both *dps* genes was confirmed by PCR using both *dps* gene-specific primer sets (Figure 1B).

**Figure 2:**
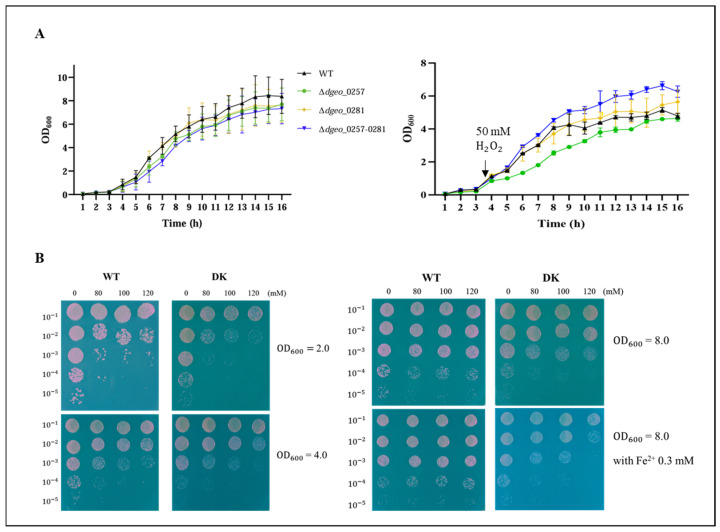
(**A**) Measurement of growth curves among WT, Δ*dgeo*_0257, Δ*dgeo*_0281, and Δ*dgeo*_0257-0281 (DK) mutants in TGY culture medium and 50 mM H_2_O_2_-treated at OD_600_ = 1.0. (**B**) Viability test between WT strain and DK mutant under different concentrations of H_2_O_2_ treatment with 80, 100, and 120 mM on three early exponential (OD_600_ = 2.0), mid-exponential (OD_600_ = 4.0), and stationary (OD_600_ = 8.0, absence or presence of 0.3 mM Fe^2+^ ion) phases. The H_2_O_2_-treated cells were diluted to 10^−5^, and 5 µL samples were spotted onto TGY medium and incubated for 2 days.

The growth rates of the single and Δ*dps*-DK mutants were compared to the wild-type (WT) strain. All mutant strains exhibited a slightly delayed onset of growth (by approximately 1 h) or a lower maximum saturation level compared to WT strain (Figure 2A). However, the absence of *dps* genes did not significantly affect growth in the TGY medium under H_2_O_2_-free conditions, consistent with observations in *D. radiodurans* [32]. This suggests that neither *dps* gene is essential for growth under these conditions. While the Δ*dps3* mutant showed delayed growth under 50 mM H_2_O_2_ conditions, Δ*dps*-DK mutant exhibited a better growth rate than the WT strain.

To evaluate the viability of Δ*dps*-DK mutant under oxidative stress, cells were treated with varying concentrations of H_2_O_2_ (80, 100, and 120 mM) for 1 h at early exponential (optical density at 600 nm, OD_600_ = 2.0), mid-exponential (OD_600_ = 4.0), and stationary (OD_600_ = 8.0) growth phases. The Δ*dps*-DK mutant exhibited similar viability to WT strain under these conditions (Figure 2B). However, the Δ*dps*-DK mutant was more sensitive than WT strain under conditions promoting Fenton reactions (0.3 mM Fe^2+^ and H_2_O_2_), a finding consistent with the generally reduced oxidative stress resistance of *dps*-deficient mutants.

To further examine the sensitivity of the Δ*dps*-DK mutant to iron, the minimum inhibitory concentration (MIC) and minimum bactericidal concentration (MBC) of iron were determined. Both WT strain and Δ*dps*-DK mutants exhibited growth inhibition with 1.5 mM iron as the MIC. However, while WT cells were killed when the iron concentration reached 12 mM, the Δ*dps*-DK mutant exhibited a lower MBC of 6 mM, indicating increased bactericidal sensitivity to iron. To precisely determine the complete cell death time (i.e., absence of colony-forming units) under the MBC of Fe^2+^, we performed a time-kill assay. The WT strain exhibited complete cell death after 9 h of exposure. In contrast, the Δ*dps*-DK mutant showed complete cell death significantly earlier, after just 6 h of exposure. These results indicate that the loss of both *dps* genes in *D. geothermalis* confers enhanced susceptibility to Fe^2+^ ions. This heightened susceptibility was observed even though the Δ*dps*-DK mutant retained ferritin and bacterioferritin-like proteins, which are typically involved in iron homeostasis and the scavenging of free iron ions to mitigate oxidative stress.

### 2.2. Transposition of IS Elements in the dps-DK Mutant

**Figure 3:**
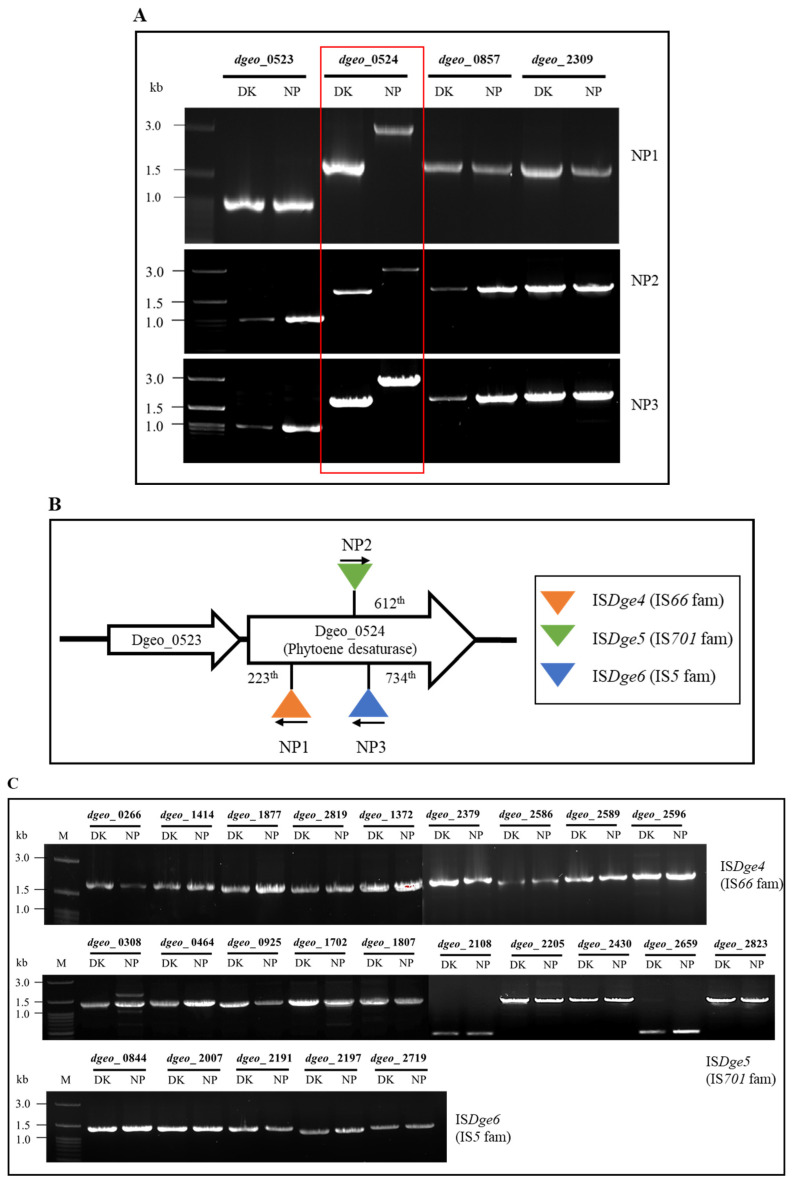
(**A**) PCR detection of IS transposition in four carotenoid biosynthesis genes at OD_600_ = 4.0 following treatment with 80 mM H_2_O_2_ in NP1 and NP2 colonies and at OD_600_ = 8.0 following treatment with 80 mM H_2_O_2_ plus 0.3 mM Fe^2+^ in NP3. All IS elements were transposed into the *dgeo*_0524 gene. The red box indicates size changes due to IS integration. (**B**) Confirmation of IS typing on *dgeo*_0524 through DNA sequencing analysis. NP1 has an IS*Dge4* of the IS*66* family, NP2 has an IS*Dge5* of the IS*701* family, and NP3 has an IS*Dge6* of the IS*5* family. The IS*Dge4* of the IS*66* family was detected for the first time on active transposition. The arrows indicate the transcriptional direction of Tpases. (**C**) Detection of IS elements transposition action mode; as genomic loci with IS*Dge4*, IS*Dge5*, and IS*Dge6* distribution still existed. Its means that they have a “copy-and-paste” transposition action mode.

Following treatment of the Δ*dps*-DK mutant at OD_600_ = 4.0 with 80 mM H_2_O_2_, non-pigmented (NP) colonies were observed on TGY plates after two days. PCR analysis of four carotenoid biosynthesis genes revealed an extended product for *dgeo*_0524, which was determined to contain an IS element (IS*Dge4*) from the IS*66* family inserted at the 223th nucleotide position in NP1 colony and another IS element (IS*Dge5* of the IS*701* family) inserted at the 612th locus of *dgeo*_0524 with the same transcriptional direction in NP2 colony (Figure 3A,B). The IS*Dge4* had incomplete identical TIRs (5′-GTGACTACTCAGCA and 5′-GTAACTGCTCAGCA) and a 449-amino-acid-long Tpase gene (Figure S1). A direct repeat (DR; 5′-CCTCGATG) sequence occurred in the DNA integration event. Thus, IS*Dge4* was classified as a member of the IS*Bst12* group, which contains a single ORF within the IS*66* family, although the family typically includes two ORFs [33,34,35]. Further experiments using a Fenton reaction-inducing condition (80 mM H_2_O_2_ and 0.3 mM Fe^2+^) at OD_600_ = 8.0 identified a third IS element (IS*Dge6*) inserted at the 734th position of *dgeo*_0524 with the opposite transcriptional orientation in NP3 colony.

In our previous studies of gene-disrupted mutants, both IS*Dge5* and IS*Dge6* were detected under various external stress conditions, such as gamma irradiation, H_2_O_2_, and DBD plasma, with targets including Δ*dps3*, Δ*dgeo*_1985-1986 as a cystine importer, Δ*dgeo*_2840 and Δ*dgeo*_1692 as LysR family regulators, and Δ*dgeo*_0606 as a sigma factor [29,30,31,36,37]. Their TIR and DR sequences were identical to each IS element.

The genome contains nine copies of IS*Dge4* element, 10 copies of IS*Dge5* element, and five copies of IS*Dge6* element positioned in the genomic loci. All of these loci were still maintained, meaning that these all three IS elements have a “copy-and-paste” action mode in transposition (Figure 3C). These findings indicate that oxidative stress promotes the active transposition of IS*Dge4*, IS*Dge5*, and IS*Dge6* in the Δ*dps*-DK mutant. Notably, IS*Dge4* transposition was identified for the first time in this study.

### 2.3. Transcriptomic Analysis of Δdps-DK Mutant

**Figure 4:**
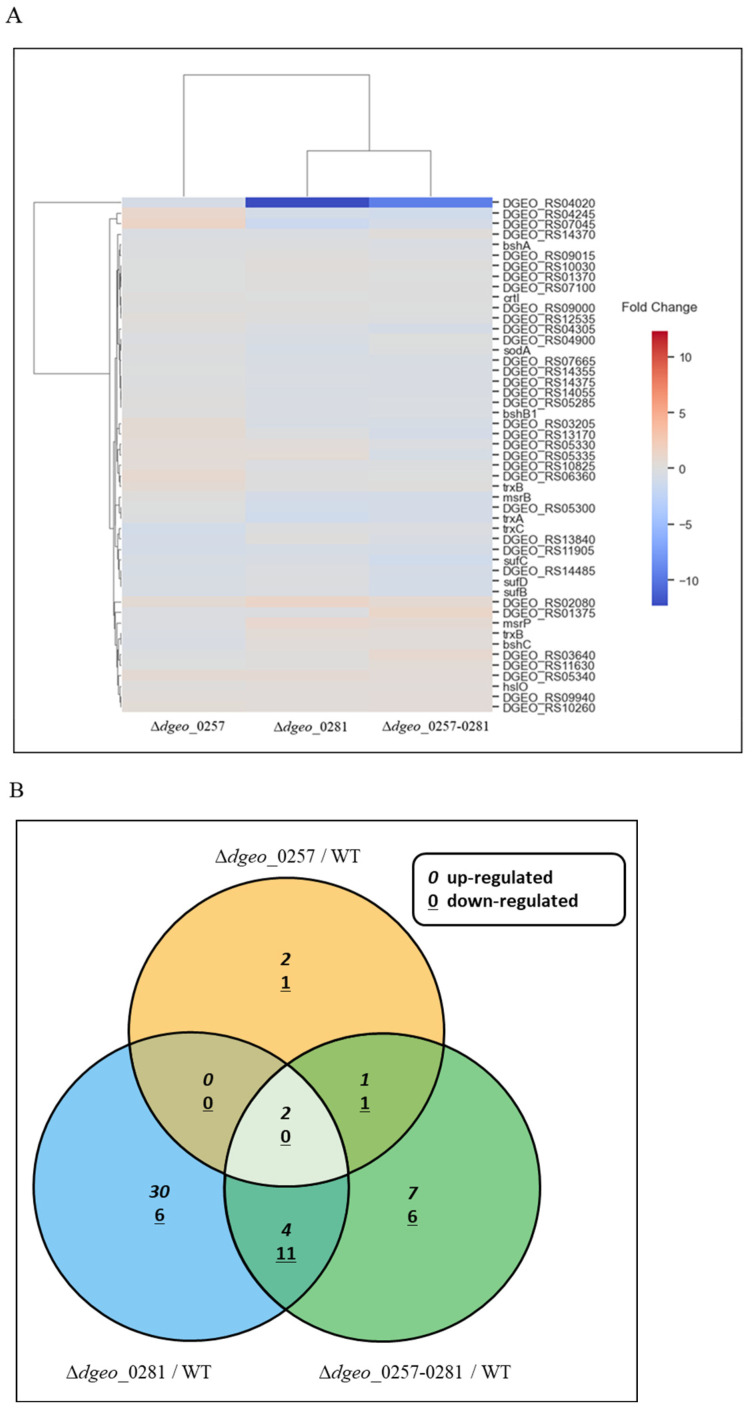
Heatmap and Venn diagram illustration of gene expression changes through transcriptomic analysis. (**A**) Heatmap of up- and down-regulated oxidative stress response-related genes in the single and DK mutants, selected based on fold changes relative to the WT strain in H_2_O_2_-absent condition (Table S1). (**B**) Venn diagram of up-regulated, contra-regulated, and down-regulated genes in Δ*dps*-single and Δ*dps*-DK mutants compared to WT under H_2_O_2_ absent condition (≥2.0-fold for up-regulated and ≤0.5-fold for down-regulated, *p* < 0.05 value). The list of target genes is provided in the supplementary data (Table S2).

RNA-seq analysis was conducted to evaluate oxidative stress response-related gene expression profiles in the WT strain, both single Δ*dps*-deficient mutants, and the Δ*dps*-DK mutant under H_2_O_2_-untreated conditions (Figure 4A; Table S1). In contrast to the general nonspecific DNA-binding function of Dps, there is specific gene regulation in the Δ*dps*-single or Δ*dps*-DK mutants (Figure 4B). It exhibits the upregulated and downregulated genes among both single Δ*dps*-deficient mutants and the Δ*dps*-DK mutant based on WT strain under the H_2_O_2_-untreated conditions.

In the absence of H_2_O_2_, the *dgeo*_1459 gene (annotated as a DUF421 family protein) was upregulated more than 25-fold in all Δ*dps*-deficient mutants (Table S2).

### 2.4. Expression Analysis of Selected Oxidative Stress Response Genes by qRT-PCR

**Figure 5:**
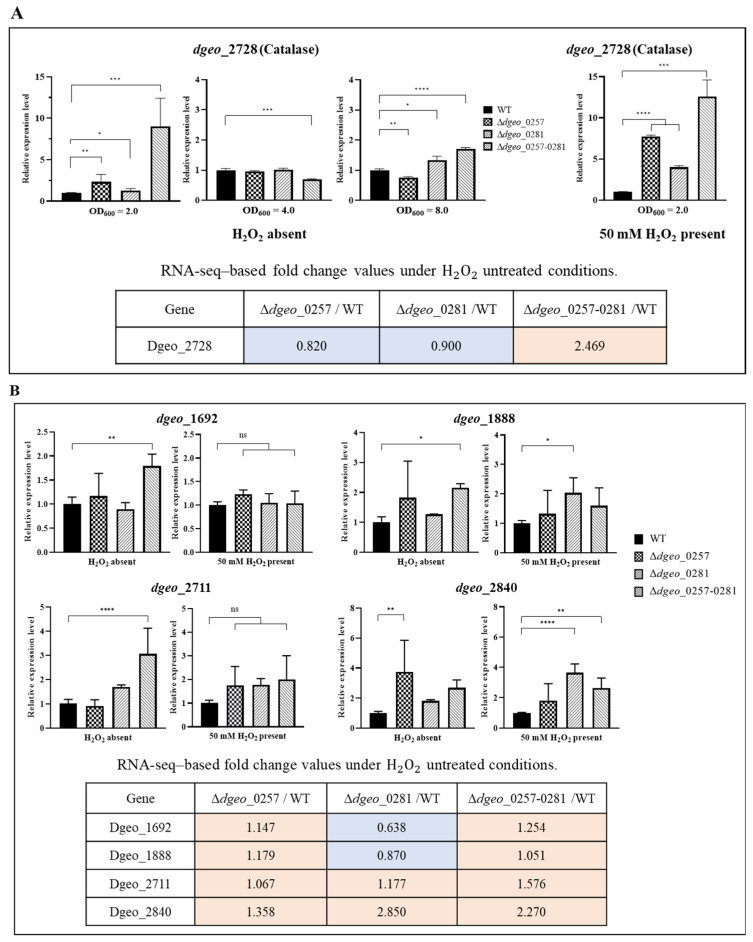

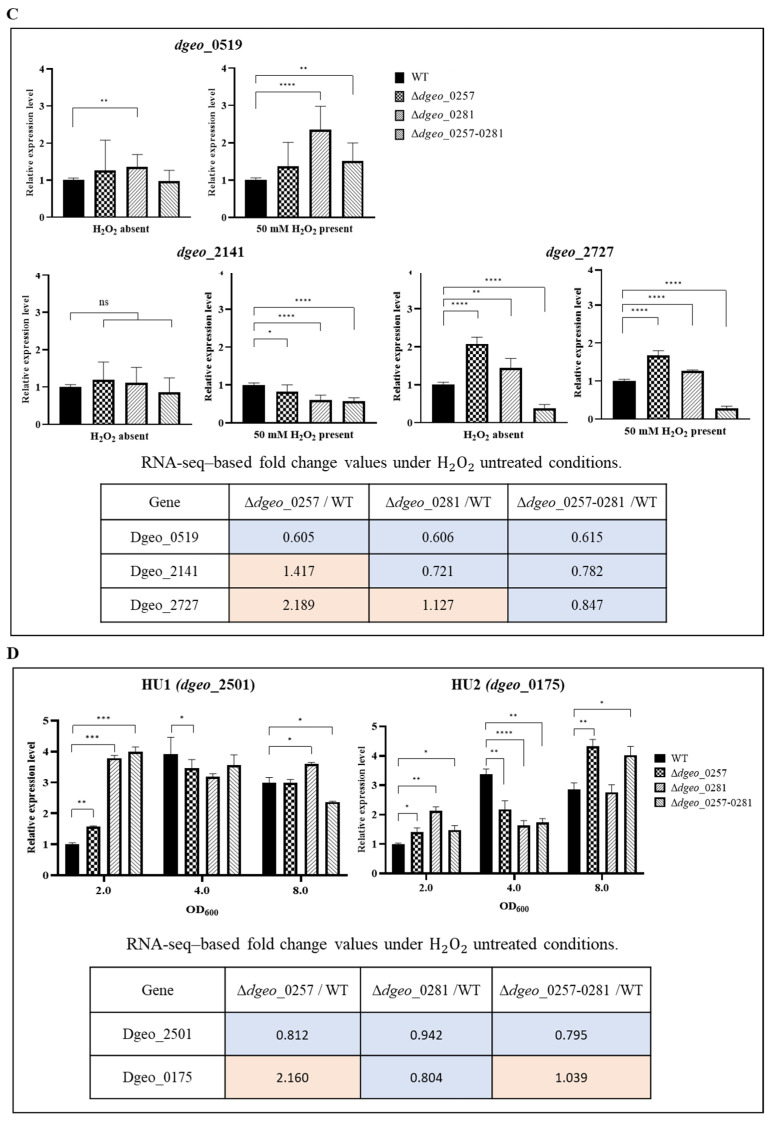
qRT-PCR confirmation of some oxidative stress-related genes expression levels. (**A**) The single catalase gene *dgeo*_2728 was analyzed in the WT strain, both single Δ*dps*-deficient mutants, and a Δ*dps*-DK mutant at the OD_600_ = 2.0, 4.0, and 8.0 growth phases. At OD_600_ = 2.0, 50 mM H_2_O_2_ treatment was applied. (**B**) The expression levels of four LysR family regulators, *dgeo*_1692, *dgeo*_1888 (OxyR), *dgeo*_2711, and *dgeo*_2840, measured in the presence or absence of 50 mM H_2_O_2_. (**C**) Three Fur family regulators, *dgeo*_2141, *dgeo*_2727, and *dgeo*_0519, were testified in the presence or absence of 50 mM H_2_O_2_. (**D**) Both HU proteins, HU1 of *dgeo*_2501 and HU2 of *dgeo*_0175, were measured in three growth phases because HU proteins are known to be dominant DNA stabilizers and one of the NAPs in *Deinococcus* strains. Statistical significance was assessed using Student’s *t*-test with Prism™ version 9.0 software, with thresholds of *p* < 0.05 (*), *p* < 0.01 (**), *p* < 0.001 (***), and *p* < 0.0001 (****). ‘ns’: indicates non-significant. The transcriptomic data for expressional fold changes were supported in Table S1.

To confirm the expression levels of several anti-oxidative stress genes, at least three iterations of a qRT-PCR assay were performed. The selected genes were catalase, LysR family regulators included OxyR as a global activator for catalase, and Fur family regulators for general antioxidation response, and two HU genes as a chromosome-stabilizing NAP. The expression levels of qRT-PCR assay were compared to the fold-changes values from transcriptomic assays among single or double *dps*-disrupted mutant strains based on WT strain under H_2_O_2_ untreated conditions (Figure 5).

Catalase expression (*dgeo*_2728) was analyzed across the WT strain, single Δ*dps*-deficient mutants, and the Δ*dps*-DK mutant. All mutants displayed at least 2-fold higher catalase expression at OD_600_ = 2.0 compared to WT strain (Figure 5A). The Δ*dps*-DK mutant exhibited higher catalase expression levels compared to the Δ*dps1.* Furthermore, upon treatment with H_2_O_2_, it shows a greater difference in expression levels than each of the single mutants.

The *D. geothermalis* genome contains four genes encoding LysR family regulators, including *dgeo*_1888 (OxyR). All LysR regulators were induced by more than 2-fold in the *dps*-DK mutant when H_2_O_2_ was absent. However, under 50 mM H_2_O_2_ treatment, LysR family members were not induced, except for *dgeo*_2840 in the Δ*dps1* and Δ*dps*-DK mutants (Figure 5B).

The *D. geothermalis* genome contains three Fur family regulators that contribute to the anti-oxidative response by mediating ferric ion uptake and maintaining intracellular iron homeostasis. Therefore, we confirmed the expression levels of the three Fur family members (*dgeo*_0519, *dgeo*_2141, and *dgeo*_2727) using qRT-PCR assays. The expression of *dgeo*_2141 was not affected by the presence or absence of 50 mM H_2_O_2_. *dgeo*_0519 was slightly induced in all Δ*dps*-deficient mutants under 50 mM H_2_O_2_ treatment. Interestingly, *dgeo*_2727 was induced by more than 2-fold in the Δ*dps3* mutant in the absence of H_2_O_2_ and slightly less than 2-fold when H_2_O_2_ was present. However, the expression level of *dgeo*_2727 was drastically downregulated in the Δ*dps*-DK mutant to less than 0.5-fold of WT strain, regardless of the presence or absence of 50 mM H_2_O_2_ (Figure 5C).

The main nucleoid-associated protein HU in *Deinococcus* was induced in a growth phase-dependent manner. In *D. geothermalis*, HU1 was strongly induced during the early exponential phase (OD_600_ = 2.0) under Δ*dps*-deficient conditions in the Δ*dps1* and Δ*dps*-DK mutants, but its expression levels were similar to that of the WT strain during the late exponential and stationary phases. HU2 was specifically induced in the Δ*dps3* strain and

Δ*dps*-DK mutants at the stationary phase (Figure 5D). In light of previous results showing that each single *dps* exhibits different expression patterns depending on the growth phase, this finding suggests a correlation between HU1 and Dps1 in the early exponential phase and between HU2 and Dps3 in the stationary phase.

### 2.5. Expression Analysis of Selected Tpases and the Upregulated Gene by qRT-PCR

**Figure 6:**
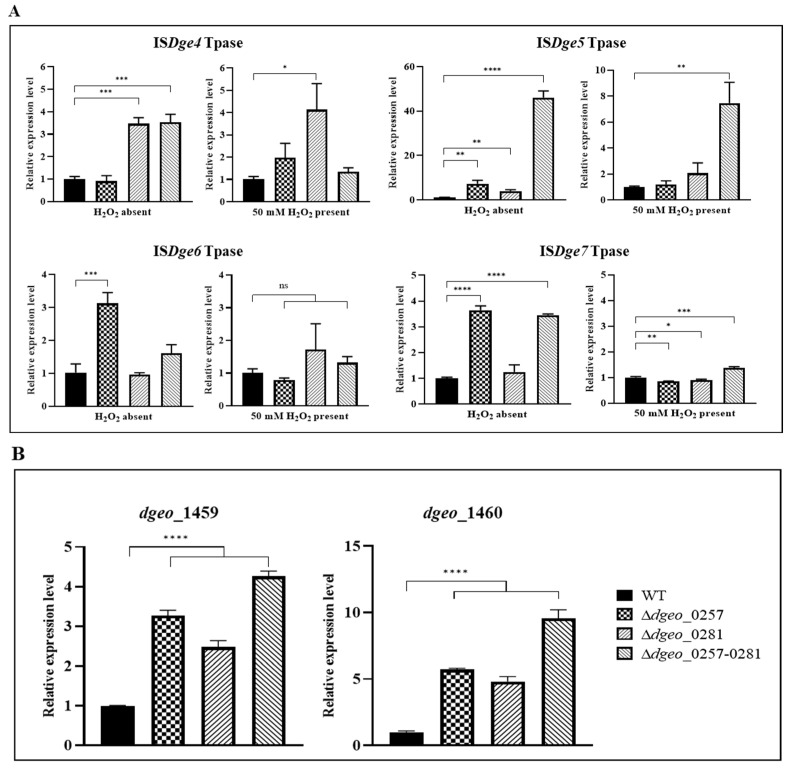
qRT-PCR confirmation of selected Tpases and the upregulated gene expression levels. (**A**) The expression levels of active transposed IS elements—IS*Dge4*, IS*Dge5*, and ISD*ge6*—and a control Tpase of IS*Dge7* were measured in the presence or absence of 50 mM H_2_O_2_. (**B**) The *dgeo*_1459–1460 gene cluster, which was the most highly upregulated among both Δ*dps*-single mutants and Δ*dps*-DK mutant. Statistical significance was assessed using Student’s *t*-test with Prism™ version 9.0 software, with thresholds of *p* < 0.05 (*), *p* < 0.01 (**), *p* < 0.001 (***), and *p* < 0.0001 (****).

The Tpases of the actively transposed IS*Dge4*, IS*Dge5*, and IS*Dge6* were expressed in the *dps*-deficient mutants (Figure 6A). The undetected Tpase of IS*Dge7* also exhibited induction mode at the absence of H_2_O_2_ in the Δ*dps3* and Δ*dps*-DK mutants. Thus, the relationship between Tpase induction and active transposition was not exactly matched yet.

In RNA-seq, the *dgeo*_1459–1460 gene cluster showed the most striking difference. This was further validated by qRT-PCR, which demonstrated at least a 4-fold difference in expression compared to the WT strain (Figure 6B). These results highlight the stringent regulatory role of *dps* genes on *dgeo*_1459–1460 expression in *D. geothermalis*.

Therefore, the Δ*dps*-deficient mutants exhibited distinct expression patterns of specific target genes. These genes included catalase, an upregulated hypothetical protein, other NAPs such as HU1 and HU2, and several oxidative stress-related transcriptional regulators (e.g., members of the LysR and Fur families). Furthermore, altered expression of selected Tpase genes was observed, correlating with the activity of both actively transposing and untransposed IS elements.

## 3. Discussion

*D. geothermalis*, a slightly thermophilic and radiation-resistant bacterium, possesses two Dps proteins: Dps1, a conserved homolog, and Dps3. Single mutants deficient in either Dps gene not only exhibit reduced maximal growth rates and lower viability under H_2_O_2_ stress but also demonstrate growth phase-dependent compensatory responses from the remaining Dps protein [18]. In this study, the Δ*dps*-DK mutant showed similarly reduced growth rates compared to the single mutants, indicating that neither Dps protein is essential for cell growth, consistent with observations in *D. radiodurans* [32]. This supports the hypothesis that Dps proteins are not critical for nucleoid compaction or cell viability under irradiation stress, with these roles likely being fulfilled by HU proteins in *Deinococcus* species [38,39]. These findings contrast with *E. coli*, where Dps and HU function together as major NAPs in a multiphasic complex [39,40]. Nevertheless, Dps proteins still have roles in nucleoid compaction under stress conditions but do not inhibit initiation of transcription by RNA polymerase, in contrast to other bacterial NAPs such as HU, FIS, and H-NS in *E. coli* [41]. The gene expression levels of the DNA stabilizer HU, the anti-oxidative response regulators of the LysR, and Fur families, and the enzyme catalase were measured using qRT-PCR in Δ*dps*-deficient mutants, but their expression levels were not significantly affected; less than 2-fold changes were found in transcriptomic analysis.

Despite this, our RNA-seq analysis of single and Δ*dps*-DK mutants revealed several *dps*-dependent differentially expressed genes (Figure 4; Tables S2 and S3). The gene cluster *dgeo*_1459–1460, encoding a DUF421 domain-containing protein and a hypothetical protein, exhibited significant upregulation (>25-fold) in all Δ*dps*-deficient mutants under H_2_O_2_-absent conditions (Figure 6B). Their expression sharply decreased in the presence of H_2_O_2_, suggesting that these genes may contribute to stress responses and cell wall synthesis, as they are located near cell wall synthesis genes [42]. In addition, the Δ*dps*-DK mutant exhibited slightly better growth than the WT strain at 60 °C, while the *dgeo*_1459–1460 gene cluster was overexpressed due to the 60 °C heat shock, as identified by qRT-PCR detection. Thus, physiological and phenotypical changes are apparent and point to the need for further experiments.

Interestingly, although a *dps*-deleted mutant of *D. wulumuqiensis* R12 exhibited reduced catalase expression, with a slight reduction in catalase 1 and a drastic reduction in catalase 2 [16], the single catalase gene *dgeo*_2728 in *D. geothermalis* was induced in Δ*dps*-deficient mutants in a growth phase-dependent manner. However, when 50 mM H_2_O_2_ was present at OD_600_ = 2.0, the expression level of the catalase gene was drastically enhanced in the Δ*dps*-DK mutant (Figure 5A).

Acknowledging the established understanding that catalase induction is generally regulated by OxyR, a member of the LysR family, in many bacterial species, it is particularly meaningful to examine the expression patterns of LysR (OxyR) genes in *Deinococcus*. This is especially true given the unique characteristics observed in *D. radiodurans*; unlike the well-studied *E. coli* strain, its OxyR possesses a single cysteine (1-Cys) and is known to function dually as both a transcriptional activator and a repressor [43]. This makes it a critical regulatory factor, orchestrating Mn and Fe transport—essential for maintaining redox homeostasis. Therefore, within the scope of our study, we systematically examined the expression levels of LysR family members in *D. geothermalis* (which encompasses four such genes: *oxyR* (*dgeo*_1888), *dgeo*_2840, *dgeo*_1692, and *dgeo*_2711). Our aim was to investigate and understand the physiological changes in gene expression under specific conditions, thereby shedding light on their potential roles in adaptation and stress response (Figure 5B). The single and Δ*dps*-DK mutants exhibited different expression profiles for these regulators. *dgeo*_1888 (OxyR) and *dgeo*_2840 were slightly induced (2–3 fold) in the *dps1*-deficient mutant under 50 mM H_2_O_2_ treatment. However, *dgeo*_1692 and *dgeo*_2711 expression levels were unaffected in the Δ*dps*-DK mutant.

Interestingly, the Fur-like gene *dgeo*_2727 was strictly downregulated in the Δ*dps*-DK mutant (Figure 5C). A *dgeo*_2727-disrupted mutant exhibited strong catalase induction (manuscript in preparation). The regulatory relationship between *dps* deficiency, Fur downregulation, catalase induction, and LysR family expression remains unclear. Therefore, the functional roles of Dps proteins in gene regulation appear to differ significantly among antioxidation response-related genes and *Deinococcus* species.

This study confirmed the active transposition of IS elements, particularly under oxidative stress conditions in the Δ*dps*-DK mutant. For instance, IS*Dge4* (IS*66* family) transposed into *dgeo*_0524 encoding phytoene desaturase following H_2_O_2_ treatment. Unlike other IS*66* family members, IS*Dge4* employs a “copy-and-paste” mechanism and possesses distinct terminal inverted repeat (TIR) sequences that are not perfectly identical, distinguishing it as a subtype of the IS*Bst12* group (Figure S1) [28,33,34,35]. This discovery provides insight into the transposition mechanisms of IS*66* family elements, which remain insufficiently characterized.

Additionally, IS*Dge5* (IS*701* family) and IS*Dge6* were actively transposed under similar conditions but were not detected in the Δ*dps3* mutant, suggesting that Dps3 may influence their mobility. These observations highlight a novel regulatory role of Dps protein in modulating IS element mobility under oxidative stress. The results suggest that Dps proteins are non-essential for growth but are critical for stress responses and genome stability in *D. geothermalis*. While the mechanisms linking Dps deficiency to IS transposition and gene regulation remain unclear, this study indicates that Dps proteins influence oxidative stress responses by regulating IS element activity and gene expression.

The findings also underscore the unique stress adaptation strategies in *Deinococcus* species, where HU proteins appear to compensate for roles typically performed by Dps proteins in other bacteria. Future work should therefore focus on elucidating the complex regulatory pathways that interconnect Dps proteins, the oxidative stress response network, and the contribution of IS element transposition. A particular emphasis should be placed on understanding the functional mechanisms involving the Fur family within these pathways. These efforts will help to better understand the genomic plasticity and resilience of *Deinococcus* species.

## 4. Materials and Methods

### 4.1. Bacterial Strains and Growth Conditions

*D. geothermalis* DSM 11300^T^ (KACC 12208) was obtained from the Korean Agricultural Culture Collection (KACC, Wanju-gun, Jeollabuk-do, Republic of Korea). *D. geothermalis* strains were cultivated in TGY medium (1% tryptone, 0.5% yeast extract, and 0.1% glucose) at 48 °C. The single Δ*dps* gene-deficient mutants (Δ*dgeo*_0257 and Δ*dgeo*_0281) and the Δ*dps*-DK mutant strain were created in previous works and in this study, respectively [29,31].

### 4.2. Construction of the dps-DK Mutant

Construction of the Δ*dps*-DK mutant was performed through homologous recombination, utilizing chloramphenicol resistance for selective purposes. To achieve targeted disruption of the *dps1* gene, the recombinant plasmid pKatCAT_dgeo_0281 was engineered. This plasmid contained the chloramphenicol acetyltransferase (*cat*) gene, which was precisely inserted between approximately 1 kb homologous sequences corresponding to the upstream and downstream regions of the *dps1* gene, and was capable of replication in *E. coli* [44]. This target gene disruption cassette was then transformed into the kanamycin-resistant Δ*dps3* mutant strain. Upon transformation with this plasmid, selection on chloramphenicol-containing medium enabled homologous recombination, leading to the successful replacement of the target *dps1* gene with the *cat* gene. The resulting Δ*dps*-DK mutant was selected on TGY agar plates supplemented with 8 µg/mL kanamycin and 3 µg/mL chloramphenicol, and incubated at 48 °C for two days. Correct integration of the *cat* gene was confirmed by PCR analysis using primers designed to amplify a region outside the integration site from extracted genomic DNA.

### 4.3. Measurement of Viability and Growth Patterns

*D. geothermalis* WT and mutant strains were cultured in 50 mL of TGY broth. Growth curves were generated by measuring OD_600_ at hourly intervals over 21 h. For the viability assay under H_2_O_2_ treatment, cells were harvested at the early exponential (OD_600_ = 2.0), mid-exponential (OD_600_ = 4.0), and stationary (OD_600_ = 8.0) growth phases. Harvested cells were resuspended in 0.9% NaCl, normalized to OD_600_ = 2.0, and treated with varying final concentrations of H_2_O_2_. After 1 h of incubation, cells were serially diluted tenfold, and 6 µL aliquots were spotted onto TGY agar plates and incubated at 48 °C for two days.

### 4.4. MIC and MBC Measurement

To test the susceptibility of *D. geothermalis* WT and Δ*dps*-disrupted mutants to H_2_O_2_, the broth microdilution method was used [44]. The bacteria were cultured to an OD_600_ of 2.0, subsequently diluted to 1:1000, and 50 µL of the diluted suspension was dispensed into each well of a 96-well microtiter plate. After preparing a two-fold serial dilution series to determine the concentration range, 100 µL of each dilution was added, and the plates were incubated for 16 h at 48 °C. The MIC was determined by measuring the OD_600_ using a Synergy HTX multi-mode reader (BioTek, Seoul, Republic of Korea). The MBC was determined as the lowest concentration at which no colony formation was observed after spreading the contents of each well onto agar plates and incubating for an additional 2 days. All experiments were performed in triplicate. Additionally, a time-kill assay was conducted in the presence of 12 mM of FeCl_2_, and samples were analyzed after incubation periods of 0, 3, 6, 9, and 12 h, following the methodology of Kim et al. [45].

### 4.5. Detection of Non-Pigmented Colonies and IS Typing

Non-pigmented mutants were selected by exposing cells to H_2_O_2_ concentrations of 50, 80, and 100 mM for 1 h. Diluted samples (10^−5^) were spread on TGY agar plates and incubated for two days. White colonies were streaked twice on fresh plates for isolation. PCR analysis using primers targeting carotenoid biosynthesis genes (*dgeo*_0523 and *dgeo*_0524, encoding phytoene synthase and phytoene desaturase, respectively) confirmed the absence of pigmentation [46]. Enlarged PCR products were sequenced to identify the types of IS elements. Transposition activity was analyzed using primers specific to the IS elements detected in the genome, which contains 19 types across nine families with a total of 73 copies [36].

### 4.6. Transcriptomic Analysis by RNA-seq

The WT strain, single Δ*dps*-deficient mutants, and the Δ*dps*-DK mutant were cultured to OD_600_ = 2.0, 50 mM H_2_O_2_ treated samples were prepared at the same OD value for 1 h, and total RNA was extracted using the Easy RNA extraction kit (Qiagen, Hilden, Germany). Library Preparation and Sequencing: The preparation of sequencing libraries from bacterial RNA was meticulously carried out using the CORALL RNA-Seq V2 Library Prep Kit (LEXOGEN, Inc., Wien, Austria). To ensure data quality and relevance, ribosomal RNA (rRNA) was effectively depleted using the RIBO COP rRNA depletion kit (LEXOGEN, Inc., Wien, Austria) from the same manufacturer. Following rRNA depletion, the resulting RNA was utilized for cDNA synthesis and subsequent shearing, strictly adhering to the manufacturer’s guidelines. For sample identification and multiplexing, indexing was performed with Illumina indexes ranging from 1 to 12. An enrichment step, crucial for library amplification, was conducted via PCR. For quality assessment, the prepared libraries were evaluated for their mean fragment size using the Agilent 2100 bioanalyzer, specifically with the DNA High Sensitivity Kit. Quantification of the libraries was then precisely determined using a dedicated library quantification kit in conjunction with a Step One Real-Time PCR System (Life Technologies, Inc., Carlsbad, CA, USA). Finally, high-throughput sequencing was performed on a NovaSeq 6000 instrument (Illumina, Inc., San Diego, CA, USA), employing a paired-end 100 sequencing strategy. Gene expression levels were quantified by read counts, and differentially expressed genes (upregulated or downregulated) were identified by comparing WT and mutant strains under both control and H_2_O_2_-treated conditions.

Data Analysis: The initial phase of data analysis involved a thorough quality control check of the raw sequencing data using FastQC (https://www.bioinformatics.babraham.ac.uk/projects/fastqc, accessed on 1 October 2025) [47]. Subsequently, adapters and low-quality reads were removed from the dataset with Fastp (https://github.com/OpenGene/fastp, accessed on 1 October 2025) [48], ensuring only high-quality data proceeded to the next steps. The trimmed and quality-filtered reads were then accurately mapped to the reference genome using STAR (http://code.google.com/p/rna-star, accessed on 1 October 2025) [49]. Following mapping, gene-level read quantification was performed with Salmon (https://combine-lab.github.io/salmon, accessed on 1 October 2025) [50]. For robust comparative analysis, the obtained read counts were normalized using the TMM (Trimmed Mean of M-values) + CPM (Counts Per Million) method via EdgeR v3 [51]. The RNA-seq information included read statistics, coverages, and quality scores, which was designed as a single sample-to-sample comparison between conditions (Table S3). RNA-seq was conducted using Ebiogen (Seoul, Republic of Korea), and data mining and graphic visualization for up- and downregulated genes were performed using “ExDEGA 5.0” (Ebiogen, Seoul, Republic of Korea). The RNA-seq data was deposited in GenBank under the accession number GSE284126.

### 4.7. Gene Expression Analysis by qRT-PCR

Cell walls were disrupted with phenol, and RNA was extracted using the RNeasy Mini Purification Kit (Qiagen, Hilden, Germany). DNA contamination was removed using DNase I. The RNA concentration was standardized to 1000 ng/8 µL, and cDNA was synthesized using the PrimeScript™ 1st Strand cDNA Synthesis Kit (TaKaRa, Osaka, Japan). The reaction was carried out using a dNTP mixture and 6-mer random primers according to the following protocol: 60 °C for 5 min, 4 °C for 3 min, 30 °C for 10 min, 42 °C for 60 min, and 95 °C for 5 min. During the 4 °C step, the reaction mixture included 5× buffer, RNase-free water, RTase, and RNase inhibitor. qRT-PCR was performed using TB Green^®^ Premix Ex Taq™ (TaKaRa, Osaka, Japan) on a Bio-RAD CFX96™ RT-PCR system (Bio-RAD, Hercules, CA, USA). Target gene expression was normalized to GAPDH, a stable housekeeping gene, and relative expression levels were calculated for catalase (*dgeo*_2728), the upregulated gene cluster (*dgeo*_1459 and *dgeo*_1460), three Fur family regulators (*dgeo*_2727, *dgeo*_2141, and *dgeo*_1519), two HU genes (*dgeo*_2501 for HU1 and *dgeo*_0175 for HU2), and four LysR family regulators (*dgeo*_1692, *dgeo*_1888, *dgeo*_2711, and *dgeo*_2840). Statistical significance was assessed using Student’s *t*-test with Prism™ version 9.0 software, with thresholds of *p* < 0.05 (*), *p* < 0.01 (**), *p* < 0.001 (***), and *p* < 0.0001 (****). qRT-PCR experiments were performed in triplicate.

## 5. Conclusions

In this study, Dps1 and Dps3 were shown to be non-essential for cell growth, as indicated by the physiological behavior of the Δ*dps*-DK mutant of *D. geothermalis*. Transcriptomic analysis revealed both upregulated and downregulated genes, suggesting that both Dps proteins are involved in gene expressional regulation, even though Dps proteins exhibit nonspecific DNA-binding properties. Interestingly, the *dps*-DK mutant exhibited a higher growth rate than both the WT and the *dps*-single deficient mutant under conditions with hydrogen peroxide. This phenomenon can be attributed to the increased expression level of catalase. Deletion of Dps in *D. geothermalis* cells leads to activation of catalase expression under normal conditions, though the exact mechanism remains unclear. This could potentially involve a disruption of the cellular redox balance, leading to minor activation of OxyR (which, however, does not affect the transcription of *oxyR* itself), or a direct involvement of Dps in catalase transcription regulation due to its DNA-binding ability. However, testing these hypotheses requires additional experiments with deletion of the *oxyR* gene and its homologues. Instead, a significant downregulation of the Fur gene was observed in the Δ*dps*-DK mutant. This observed interplay between Fur and catalase therefore suggests a Dps-linked network regulatory system, the complete understanding of which warrants further investigation.

The Δ*dps*-DK mutant exhibited active transposition of IS elements, including the novel detection of IS*Dge4*, a member of the IS*66* family, which contains an incomplete TIR sequence. In this study, the IS*Bst12* group of the IS*66* family was reported to exhibit active transposition for the first time. It remains unclear how IS*Dge4* transposes into other genomic loci under oxidative stress conditions. Notably, transposed IS*Dge2*, IS*Dge5*, and IS*Dge6* elements were exclusively observed to insert into *dgeo*_0524 in both the Δ*dps*-deficient mutants and the WT strain. These findings suggest that Dps proteins in *D. geothermalis*, while primarily recognized for their roles in Fe^2+^ ion accumulation and chromosome stabilization, may also be involved in the regulation of antioxidative stress response genes and the promotion of active IS element transposition. Their function could help define the contribution of IS element mobility to gene function, genome plasticity, and genomic dynamics. These findings indicate that the discovery of novel IS elements, especially those found in contexts other than simple single mutants, can contribute valuable information to the field of Dps research.

## Data Availability

The data presented in this study are openly available in GenBank [https://www.ncbi.nlm.nih.gov/genbank/, accessed on 1 July 2025] [GEO: GSE284126, PRJNA1197403].

## Supplementary Materials

The following supporting information can be downloaded at: https://www.mdpi.com/article/10.3390/ijms27031238/s1.

## Abbreviations

The following abbreviations are used in this manuscript:

Dps	DNA-protecting protein in starved cells
IS	Insertion sequence
MIC	Minimum inhibitory concentration
MBC	Minimum bactericidal concentration
Tpase	Transposase
