# Supplementary material for: Physiological Changes and Transposition of Insertion Sequences in the dps-Double-Knockout Mutant of Deinococcus geothermalis
Source: Int J Mol Sci. 2026 Jan 26;27(3):1238. doi: 10.3390/ijms27031238 (PMC12898284; doi:10.3390/ijms27031238)
Supplement: Supplementary file 1 [file ijms-27-01238-s001.zip › Figure S1.pdf]

Supplementary data

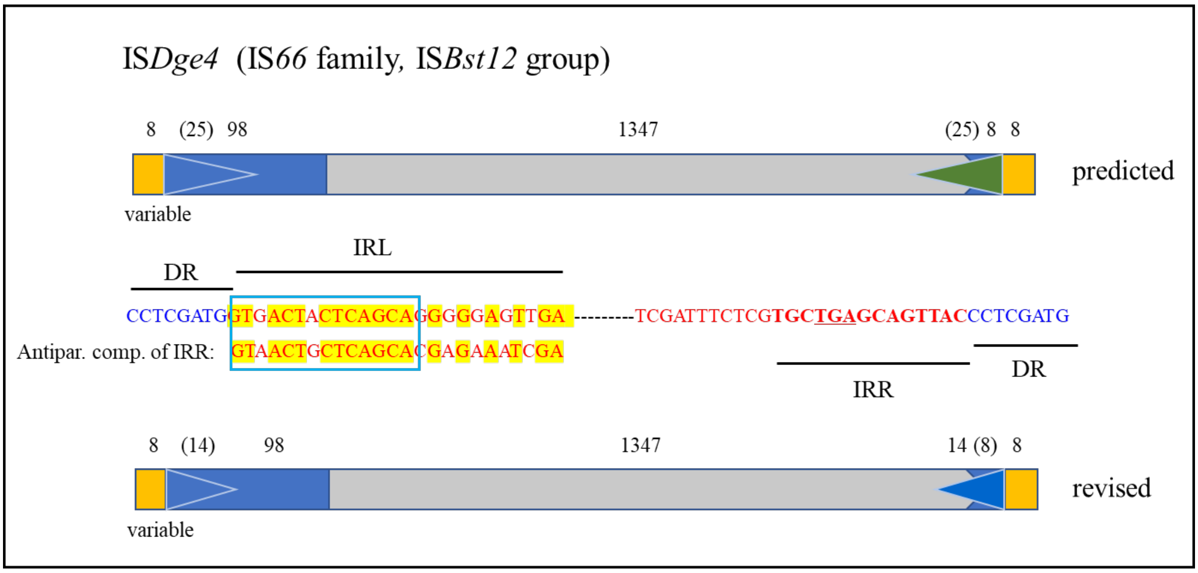

Figure S1. Scheme of IS*Dge4* of the IS*66* family as IS*Bst12* group in *D. geothermalis*. In this work, the TIR sequence of IS*Dge4* was revised into 14-lengths from previous 25-lengths following by [34].
